# Identification of dust storm origin in South –West of Iran

**DOI:** 10.1186/s40201-017-0280-4

**Published:** 2017-07-17

**Authors:** Parya Broomandi, Bahram Dabir, Babak Bonakdarpour, Yousef Rashidi

**Affiliations:** 1 Department of Chemical Engineering, Amirkabir University of Technology, Tehran, I.R Iran; 2 Environmental Sciences Research Institute, Shahid Beheshti University, Tehran, I.R Iran

**Keywords:** Iran, RDI, COMSALT, Atterberg limits, Particle size distribution, Wind erosion, Mineralogical composition, Soil and dust chemistry

## Abstract

**Background:**

Deserts are the main sources of emitted dust, and are highly responsive to wind erosion. Low content of soil moisture and lack of vegetation cover lead to fine particle’s release. One of the semi-arid bare lands in Iran, located in the South-West of Iran in Khoozestan province, was selected to investigate Sand and Dust storm potential.

**Methods:**

This paper focused on the metrological parameters of the sampling site, their changes and the relationship between these changes and dust storm occurrence, estimation of Reconaissance Drought Index, the Atterberg limits of soil samples and their relation with soil erosion ability, the chemical composition, size distribution of soil and airborne dust samples, and estimation of vertical mass flux by COMSALT through considering the effect of saffman force and interparticle cohesion forces during warm period (April–September) in 2010. The chemical compositions are measured with X-ray fluorescence, Atomic absorption spectrophotometer and X-ray diffraction. The particle size distribution analysis was conducted by using Laser particle size and sieve techniques.

**Results:**

There was a strong negative correlation between dust storm occurrence and annual and seasonal rainfall and relative humidity. Positive strong correlation between annual and seasonal maximum temperature and dust storm frequency was seen. Estimation of RDI_st_ in the studied period showed an extremely dry condition. Using the results of particle size distribution and soil consistency, the weak structure of soil was represented. X-ray diffraction analyses of soil and dust samples showed that soil mineralogy was dominated mainly by Quartz and calcite. X-ray fluorescence analyses of samples indicated that the most important major oxide compositions of the soil and airborne dust samples were SiO_2_, Al_2_O_3_, CaO, MgO, Na_2_O, and Fe_2_O_3_, demonstrating similar percentages for soil and dust samples. Estimation of Enrichment Factors for all studied trace elements in soil samples showed Br, Cl, Mo, S, Zn, and Hg with EF values higher than 10.

**Conclusion:**

The findings, showed the possible correlation between the degree of anthropogenic soil pollutants, and the remains of Iraq-Iran war. The results expressed sand and dust storm emission potential in this area, was illustrated with measured vertical mass fluxes by COMSALT.

## Background

Arid and semi-arid climates covering much of Iran, make these areas vulnerable to the desertification if they are inappropriately managed. Iran has a land area about 1.64 million km^2^, including 30 provinces, with variable and often extreme climate, with extended periods of high temperatures above 40 °C and with sub-freezing temperatures as low as −20 °C [1]. Maximum rainfall reaching around 1200 mm in the north decreases to a minimum of less than 100 mm in the central region. Totally, deserts are covering approximately 20% of Iran’s area. Iran’s Bureau of Desert Affairs is classifying the greater part of the country as being arid or hyper-arid (see the Fig. 1 in [1]). It is believed that the climatic factors are the main desertification causes (The long-term average annual percipitation for arid and semi-arid regions in Iran is 141.1 mm), population, over-exploitation of water resources, and over-grazing [1]. During the last years, intensive dust storms frequencies were significantly increased in Iran, while affecting human health in the southern parts of Iran like the southwestern Khuzestan Province and the northern part of southeastern Sistan and Baluchistan Provinces [1]. Also, these storms are seriously disturbing the life of the people in these regions and putting even their breathing in trouble [2]. Goudarzi and co- workers showed about 17% of total hospital admissions, cardiovascular death, and respiratory mortality occurred when the PM10 concentrations were more than 30 μg/m^3^. Also, sum of respiratory and cardiovascular related to PM10 were 1055 and 189 cases in 2012, when the annual average concentration of PM10 was 321 μg/m^3^ in Ahvaz city [3]. In similar studies, the association between PM10 levels and daily mortality was investigated in Ahvaz city. Also, Naimabadi and co- workers investigated the relation between cytotoxicity and the risk of PM10 to human lung. The results show that due to inhalation of a higher mass concentration of airborne particles, cytotoxicity can be more severe during dust storm in compare with normal days [4–6]. Also, this natural disasters can influence the rate of internal migration in these regions. The researchers found out that, on average, natural disasters like dust storms occurrence and drought can increase the number of migrants in the affected areas [7, 8]. The number of people engaging in international migration because of climate changes in comparison with the number of people engaging in internal migration is small [8]. However, one outstanding argument is that climate change is able to affect the livelihood and incomes of people in developing countries especially result in an increase their incentives to migrate to rich countries. Two main subjects for researching in Iran are increasing the frequency of dust storm events and dust origins. Some studies based on satellite images and metrological data have been conducted to determine local dust sources. The investigations show that the substantial dust origins in Iran are Al-Howizeh/Al- Azim marshes and Sistan basin: Al-Howizeh/Al-Azim marshes are straddling the Iran-Iraq border and Sistan basin is centered at ~3 l’ N, 61.5′E [9]. Moreover, the foreign main dust sources are Iraq, Syria, Saudi Arabia and Kuwait. Specifically, Iraq is one of the main sources for dust storm in Iran. Iraq with extensive areas of sand deserts (nearly 40% of the country’s total area) should be a source of great concerns. In this country, the rate of desertification has increased because of severe drought of 1990, deforestation actions, inappropriate land use, war impacts, political instability, improper internal decision- making and dam projects in neighboring countries [10, 11]. In order to understand the role of soil consistency and variation in regional metrological parameters in susceptibility of soil to wind erosion and establishing functional remedial strategies and policies, it is regarded as necessary to study the physical, mechanical and chemical characteristics of soil and airborne dust to estimate the sand and dust storm potential of Shalamcheh region (the south-west of Iran). To the best of our knowledge, there are currently no published studies about mechanical, physical and chemical characteristics of soil and airborne dust in this area (Shalamcheh) and estimation of wind erosion capacity, but only some studies on the geochemical and mineralogical characteristics of blown dust at khoozestan province in south-west of Iran were conducted [12]. In this paper, one of the sand storm sources at the south-west of Iran in Khoozestan province was recognized and annual vertical mass fluxes were determined by COMSALT. The goal of this paper provides an overview of the current state of knowledge in this area, highlights current controversies, and identifies future research needs. Particularly, the study has focused on the following questions: (i) What are the metrological parameters of the sampling site, their changes during last 60 years and the relationship between these changes and dust storm occurrence, (ii) How to estimate the Reconnaissance Drought Index, (iii) What are the Atterberg limits of soil samples and their relation with soil erosion ability, (iv) What are chemical composition, size distribution of soil and airborne dust samples, and (v) How to estimate the vertical mass flux by COMSALT.

**Fig. 1:**
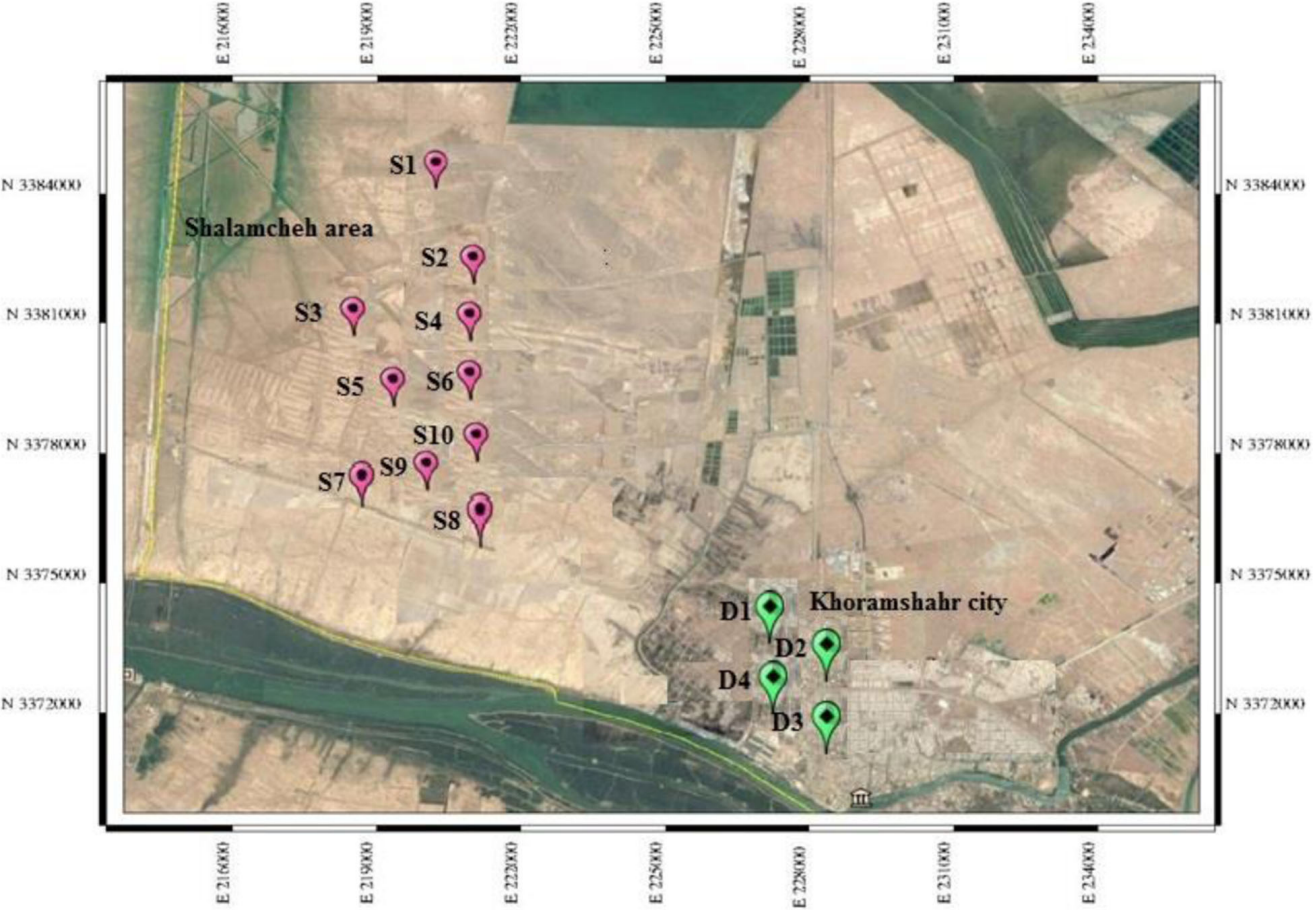
The location of Soil sampling (10 point in Shalamcheh area) and Dust sampling (4 points in Khoramshahr city) located in Khoozestan province of Iran during warm period (April–September) in 2010

## Methods

### Description of study area and sampling method

The study area, Shalamcheh, is located in the south-west of Iran in Khoozestan province (30°30′18″ N and 48°01′35″ E). Ten soil samples were collected from various surface soil points based on the US EPA test methods (Method 5035). Sampling depth was 0 to 15 cm during the warm period (April–September) in 2010, respectively (Fig. 1) [13]. Also, dust samples were taken from Khoramshahr (which is the nearest city to the studied area). In order to collect dust samples, a dry flat surface with an area of 1 m^2^ was chosen based on the dust sampling instrument developed by Menendez and co- workers in 2007 [14]. The simplest dry dust collector is consist of a glass surface (100 × 100 cm) which is covered with a 2 mm PVC mesh on top to form a rough area for trapping saltating particles. The dust collectors were placed on the roof of the buildings with 3–4 m height above the ground. Dust samples were gathered over the sampling period of 30 days during the warm period in 2010. The studied area was hit by strong dust storms during warm period in 2010 (Fig. 1). Dust particles were gathered using scraping tools adhered to the glass trays by a rubber spatula. The trays were washed before the next collection. The total number of collected dust samples was 24. It should be mentioned that dust sampling was not permitted in the studied area because it is a military and restricted area.

### Estimation of Reconnaissance Drought Index (RDI) for studied area

Different drought indices have been developed to quantify if a region experiences a drought and to categorize the severeness of the drought. The ratio between two potential evapotranspiration and aggregated quantities of rainfall is used in this method (see Table 1 in [15]). α _0_ is calculated as the coefficient of the i^th^ year in aggregated form, using a monthly time step is calculated as ${\alpha}_0^i=\frac{\sum_{j=1}^{12}{P}_{i j}}{\sum_{j=1}^{12}{P ET}_{i j}}$, where, *i* = 1(1) N and j = 1(1)12, PETij and Pij are potential evapotranspiration and rainfall of the j^th^ month of the i^th^ year and N is the total number of years of the existing data. The Normalized RDI (RDI_n_) is computed, using α (the arithmetic mean of α _0_ values) based on gathered meteorological data as, ${RDI}_n^{(i)}=\frac{\alpha_0^{(i)}}{\overline{\alpha_0}}-1$. The Standardized RDI (RDI_st_) is computed as, ${RDI}_{STK}^{(i)}=\frac{\left({y}_k^{(i)}-\overline{y^k}\right)}{\sigma_{y k}}$, where, y_k_ is the $\ln \left({\alpha}_0^{(i)}\right)$, y_k_ is the arithmetic mean and *σ*_*yk*_ is the standardized deviation [15].

**Table 1:** Initial soil and wind parameters used in COMSALT for horizontal mass flux calculation

Parameter	Value
D_pavg_ (Average particle diameter)	138 μm
U^*^(Wind shear velocity)	0.122 m/s
ρ (Particle density)	2204 kg/m^3^

### The chemical, mineralogical and mechanical analyses of soil and dust samples

#### Grain size distribution analyses

Particle size distribution analyses were conducted by using the sieve and laser particle size analysis (Analysette 22 μ Tec Plus- Fritsch) techniques [16]. In order to determine the soil texture class, USDA classification was used [17], which is based on the proportion of sand 2.0–0.05 mm, silt 0.05–0.002 mm and clay <0.002 mm particles in soil.

#### Atterberg limits determination

The Attaerberg limits were determined through using casagrande Method [18, 19]. The water content of fine grained soils at different states of consistency is introduced by Atterberg limits based on plastic limit (PL) and liquid limit (LL) and more significantly on plasticity index (PI). The plasticity index is an evaluation of the difference between the plastic and liquid limit (that is, PI = LL-PL) [17].

#### Soil organic matter

The Walkley-Black Method was used to determine soil organic matter [20]. In this method, concentrated H_2_SO_4_ and K_2_Cr_2_O_2_ are added to between 0.5 g and 1.0 g of soil samples. The solution was swirled and let to cool prior to adding water to halt the reaction. When the sample has cooled, H_3_PO_4_ was added to the digestive mix after to eliminate interferences from the ferric iron that may be present in the sample. At this point 3 or 4 drops of Ferroin indicator was added and titrate with 0.4 N FeSO_4_ to a burgundy endpoint. The organic matter content was calculated using the difference between the total volume of dichromate added and the volume titrated after reaction.

#### Mineralogy analysis

The mineralogy of collected soil and dust samples were determined using XRD (Inel- EQuniox 3000) and XRF (X unique II Rh 80kv Lif220 GeT1AP) methods.

#### Analyses of the heavy metals

The heavy metals content in the soil and dust samples were measured by using AAS (Varian- AAS- 240). To determine the concentration of heavy metals in airborne dust samples each weighting 0/5 g were digested using 60% pure HNO3 for 24 h at 80 °C. The residue was filtered using Whatman No. 42 filter paper and then diluted with 25 mL of 1% pure HNO_3_water in a volumetric flask [21].

### Enrichment factor analysis

Using EF relative to the Earth’s upper crust composition, it is possible to distinguish between the sources of elements from anthropogenic or crustal origins. Reference elements are usually Si, Al or Fe, but it is not a universal accepted rule. In this study, Si was considered as the reference element [22]. The values of EF_Si_ are very close to 1 [22]. EFs for crustal material (EF_Crust_) are measured as, ${\mathrm{E}\mathrm{F}}_{\mathrm{Crust}}=\frac{{\left[\frac{\mathrm{E}}{\mathrm{R}}\right]}_{\mathrm{Soil}}}{{\left[\frac{\mathrm{E}}{\mathrm{R}}\right]}_{\mathrm{Crust}}}$, where E is the elemental concentration, R is a reference element (R = Si for the present study) and [E/R] _Soil_ is the concentration ratio of E to R in a collected soil sample, and [E/R] _Crust_ is the concentration ratio of E to R in the Earth’s crust [23].

### Model description

Sand storms which are basically wind storms carrying sand through the air, creating a relatively low cloud near the surface. Sandstorms typically can reach heights of up to 15 m, including sand grains with mean sizes between (0.15–0.30) mm, when wind velocity exceeds 14 Km/h and last as long as wind velocity persist. When the wind exceeds a critical speed, sand grains start to move forward along with the ground surface. For higher wind velocities, sand grains in sand storm are moved by saltation process [11]. Since the saltation process plays a major role in creating dust emissions, COMSALT, which is the first physically based numerical model, is used to estimate the vertical particle mass flux in studied area. It also includes physically- based parameterization of the splashing particles. In COMSALT, the initial trajectories of saltators are calculated using the logarithmic wind profile formed by a turbulent fluid flowing over a no-slip surface [24] as, ${u}_z=\frac{u^{\ast }}{k} \ln \frac{z}{z_0}$, where k = 0.4 is the von Karman constant, u_∗_ is the friction velocity or wind shear velocity, z is the vertical distance from the surface, and ${z}_0\approx \frac{2{D}_p}{30}$ is the aerodynamic surface roughness. As in previous investigations, the wind flow is assumed to be horizontal. The initial wind profile given by logarithmic wind profile is modified because of the momentum transfer and shear stress between the wind flow and saltating particles. For simplicity, the particle motion simulation was assumed in two dimensions. The saltating particle collisions with each other, as well as the electrostatic force effects on particle trajectories were neglected in this model [24]. The effect of these processes was not important for small to medium shear velocities (i.e., u* < ~0.5 m/s) but probably became considerable for larger shear velocities [24]. Saltation simulation was studied in steady-state. Saltating particle motions are simulated using gravity force, fluid drag, fluid shear, particle spin and turbulence, respectively. Also, the turbulence effect on particle trajectories, which was often neglected by previous numerical models of saltation, was considered in COMSALT [24]. Considering horizontal mass flux as one of the principle components of Aeolian sediment flux, the initial soil and wind parameters (listed in Table 1) are used for horizontal mass flux calculation. The vertical mass flux is proportional to the horizontal mass flux and is an alternative approach to derive any formula for emitted vertical mass flux by an eroding soil (F_d_) [25]. However, having a similar scaling with u_∗_ by the amount of the kinetic energy impacting on the soil surface and saltation flux (see Table 1 and eq. (4.11) in [24]). This assumption resulted in; *F*_*d*_ = *αQ*, where α is the sandblasting efficiency, which is on the order of 10^−5^–10^−2^ m^−1^ [23] and Q is horizontal mass flux.

## Results

The annual and seasonal changes of meteorological parameters over the last 60 years were studied. Also, the correlation coefficients between the annual and seasonal dust storm occurrence and precipitation, maximum temperature, relative humidity and wind speed were calculated. Data taken from Abadan and Khoramshahr metrological station (32°.26′N, 48°.09′E), which is the nearest station to the Shalamcheh, records the climatic elements within Shalamcheh and dust storm frequencies (times of the dust storm occurrence) for the years 1951–2014. Also, the variations of meteorological parameters during warm period (April to September) and cold period (October to March) for the last 60 years were studied [26]. The annual received rainfall was about 36.8 mm, the annual average maximum temperature was 35.7 °C and the annual average relative humidity was 41% in 2010 (studied period). This area is totally bare and any vegetation cover cannot be seen.

### The dust storm occurrence in Shalamcheh

**Fig. 2:**
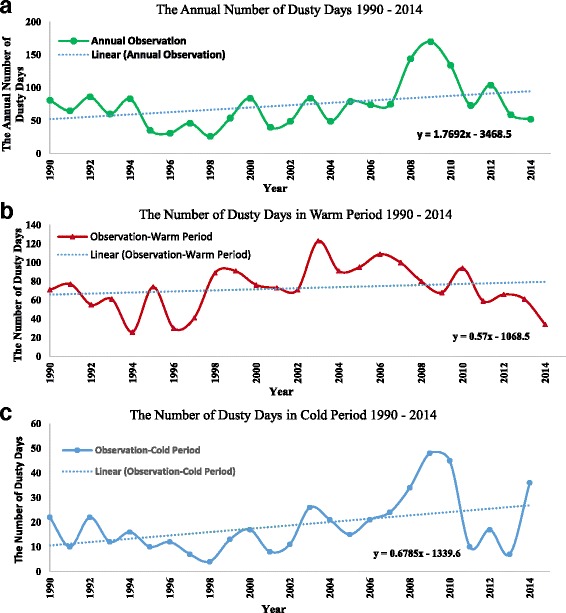
Dust storm occurrence in the studied area during (**a**) last 25 years 1990–2014, **b** during warm period, and **c** during cold period

The dust storm occurrence of about 25 years period (1990–2014) were studied. The observations indicated a considerable increase in dust storm frequencies in the studied area. The average number of dust storms in 2008, 2009 and 2010 is higher compared with the mean trend line (Fig. 2). Also, the results showed an increasing trend in dust storm occurrence during cold period (October to March). It reached to 48 days in 2009. During the warm period (April to September), the annual dust storm occurrence experienced a remarkable increase in the last decades. This value increased to 122 days in 2009 (during warm period) in the studied area. The recorded data show an average of 45 cold dust storm days per year and 89 warm dust storm days per year in 2010 (Fig. 2). Table 2 is an illustration of the occurred strongest and the most intense dust storms in the studied area during the warm period in 2010 (studied period), reduced the horizontal visibility to 1000 m and below.

**Table 2:** The most intense dust storms occurred in the studied area during the warm period in 2010

Date	Horizontal Visibility	Date	Horizontal Visibility
3/4/2010	200 m	5/24/2010	100 m
3/5/2010	800 m	6/7/2010	100 m
3/18/2010	500 m	6/8/2010	500 m
3/19/2010	700 m	6/16/2010	500 m
3/26/2010	200 m	6/23/2010	600 m
3/27/2010	200 m	6/24/2010	1000 m
4/4/2010	400 m	6/28/2010	200 m
4/5/2010	800 m	6/30/2010	500 m
4/23/2010	800 m	7/19/2010	1000 m
4/27/2010	1000 m	7/22/2010	1000 m
5/13/2010	200 m	7/29/2010	1000 m
5/14/2010	1000 m	8/4/2010	300 m
5/17/2010	600 m	8/15/2010	200 m
5/18/2010	800 m	9/10/2010	1000 m

### The annual and seasonal rainfall, maximum temperature and relative humidity in Shalamcheh

**Fig. 3:**
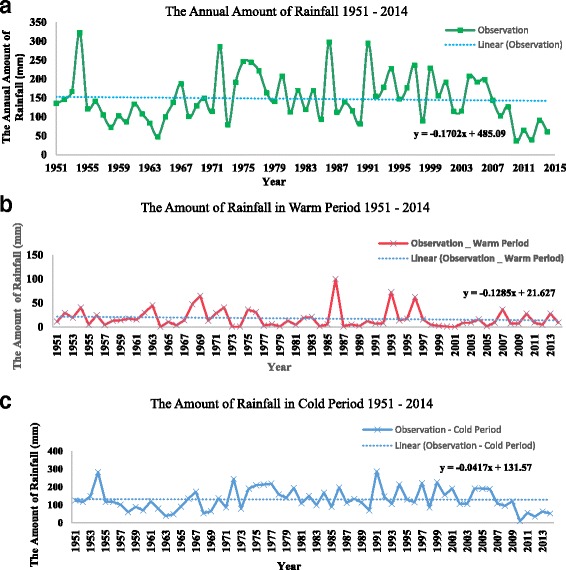
Annual amount of Rainfall in the studied area during (**a**) over last sixty years 1951–2014, **b** during warm period, and **c** during cold period

**Fig. 4:**
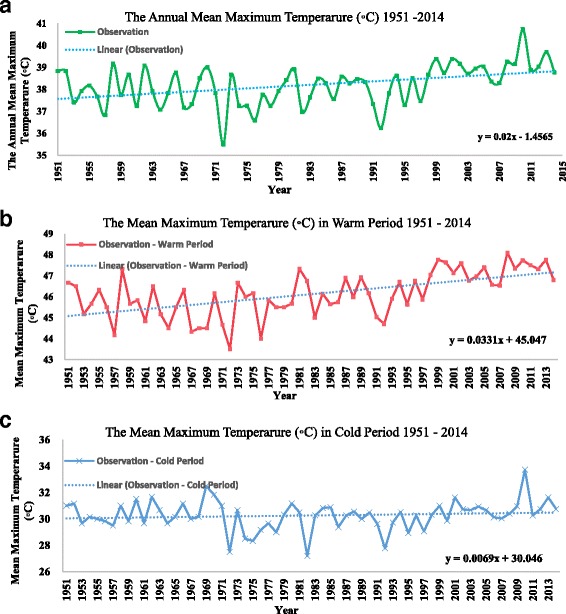
Mean Maximum Temperature (°C) in the studied area during (**a**) over last sixty years 1951–2014, **b** during warm period, and **c** during cold period

**Fig. 5:**
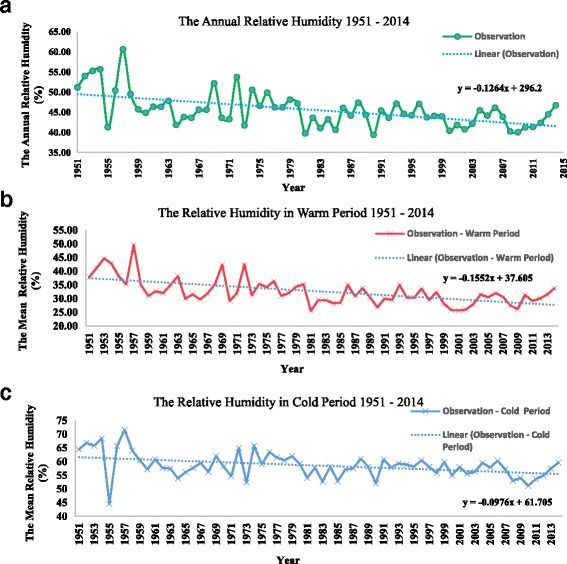
Mean Annual Relative Humidity (%) in the studied area during (**a**) over last sixty years 1951–2014, **b** during warm period, and **c** during cold period

The annual rainfall (mm), maximum temperature (°C) and relative humidity (%) of about 60 year’s period were studied. The results show a remarkable decrease in the annual rainfall. It reached to 36.8 mm in 2010. Considering the changes in precipitation during the cold season, a substantial reduction was revealed in the last 60 years (Fig. 3). It also, experienced a reduction during warm period in the last decades. The results reflected that there was a notable increase of mean average annual maximum temperature with time. In addition, the same increasing trend was detected in the seasonal studies of variations in average mean maximum temperature during cold and warm periods (Fig. 4). Also, remarkable decrease in the average mean annual Relative Humidity (in %) for 60 years intervals has been indicated. The decreasing trend of average mean relative humidity during cold and warm periods was seen between 1951 and 2014, respectively (Fig. 5).

### Wind rose plot

**Fig. 6:**
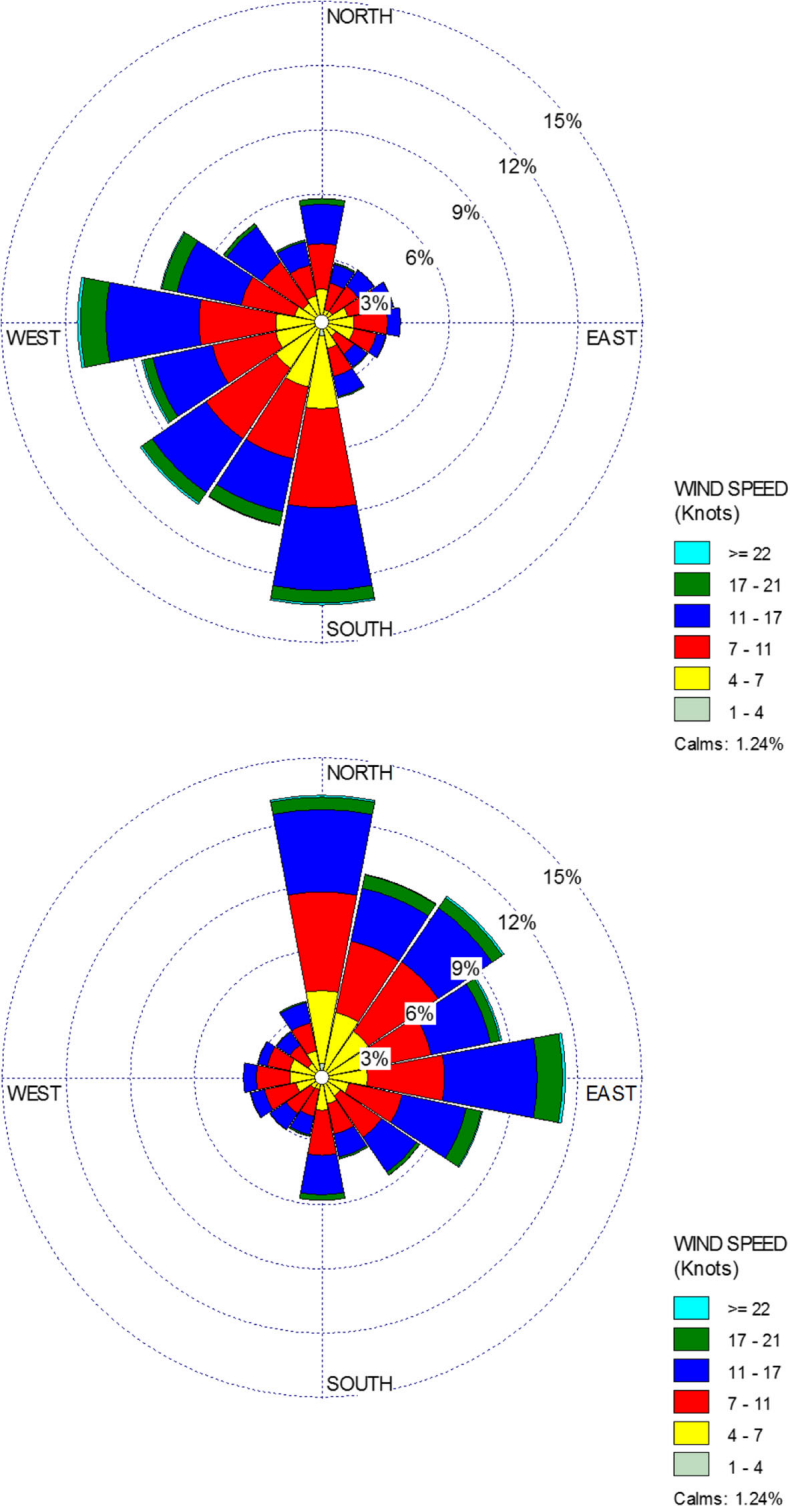
Wind Rose Plot of wind speed direction blown from South-West to North-East in the studied area

Wind rose plots are frequently used to show the distribution of wind speeds and their varying directions in the study area, based on their metrological observations. As it can be seen in Fig. 6, most of the winds blow from South-West to North-East. Also, the wind class frequency distribution indicates prevail wind class is 7–11 knots with prevail percent of 36.1%.

### Reconnaissance Drought Index (RDI) in Shalamcheh

**Fig. 7:**
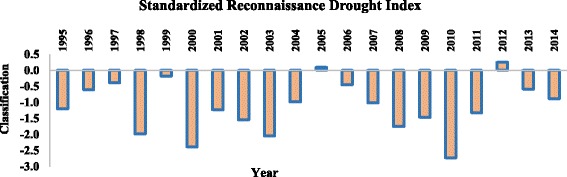
The calculated Standardized Reconnaissance Drought Index in the studied area during 1995–2014

The results show for Standardized RDI, during the years of 1995, 2001, and 2007, there was Moderately Dry Condition. Also, during the years of 1998, 2002, 2008, 2009, and 2011, there was Severe Dry Condition. While the Extreme Dry Condition was seen during the years of 2000, 2003, and 2010 (Fig. 7).

### Particle size distribution, OM content of soil Samples

Particle size analysis and respective soil textural class are reported in Table 3. The results showed that sand content was generally high in all samples. Soil textural class for soils in the studied area is mainly sandy (at least 80%) with relatively low OM (0.060%).

**Table 3:** Composition of soil texture for Case Study (based on particle size distribution analysis [16])

Sampling Point	%Clay	%Silt	%Sand	%OM	%OC
A_1_	1	13	86	0.061	0.035
A_2_	6	14	80	0.073	0.042
A_3_	5	15	80	0.069	0.04
A_4_	2	10	88	0.058	0.033
A_5_	7	20	73	0.1	0.058
A_6_	1	13	85	0.061	0.035
A_7_	3	14	83	0.065	0.037
A_8_	5	15	80	0.071	0.041
A_9_	7	16	77	0.082	0.048
A_10_	8	13	79	0.079	0.046

### Plastic limit, liquid limit and plastic index

Plastic limit of the soil samples was measured around 24% and liquid limit was 38%. The plasticity PI index (14%) as a qualitative manner classified soil samples as medium plasticity. Similarly, according to the plasticity chart classified they are classified inorganic clay, and medium compressibility [27].

### Mineralogy of soil and dust samples by using XRD and XRF analysis

The soil and dust samples were detected for major oxides and minerals by using XRD and XRF techniques, respectively. The recognized frequent minerals in soil and dust samples are; Quartz, Chlorite, Dolomite, Kaolinite, Mica, Montmorillonite, Illite, Calcite, Gypsum and Halite. Furthermore, mean major oxides content of soil and dust samples are demonstrated in Table 4. The dominant components of soil samples are Quartz and Calcite while BaO is not detected in this area. Also, the dominant components of dust samples gathered over Khoramshahr city are Quartz and Na_2_O while BaO is not detected in dust samples too.

**Table 4:** Comparison between abundance of Oxides in upper continental crust, soil samples and dust samples in studied area [43]

Oxide	% in upper continental crust	% measured in soil samples in studied area	% measured in dust samples in studied area
SiO_2_	61.5	29.2	31.9
Al_2_O_3_	15.1	4.9	6.1
Fe_2_O_3_	6.28	4.19	2.13
CaO	5.5	17.8	10.1
Na_2_O	3.2	1.82	20.9
MgO	3.7	6.3	17.7
K_2_O	2.4	1.12	1.7
TiO_2_	0.68	0.51	0.49
BaO	0.0584	---	---
MnO	0.1	0.078	0.004

### Anthropogenic soil and dust pollution

The EF calculation of soil samples show that Br, Cl, Mo, S, Zn and Hg had average EF higher than 10, were considered to originate mainly from anthropogenic origins. Also, the EFs for trace and major elements of dust samples indicated that EFs of trace elements Br, Mo, S, Zn and Hg higher than 10, showing their anthropogenic sources (Table 5).

**Table 5:** Average AAS values for trace elements of airborne dust samples and soil samples in studied area

Trace elements	Soil (ppm)	Dust (ppm)
Cl	44,000	604
S	37,200	7700
Br	93	6.2
Co	13	14
U	1.7	5.5
Cr	156	229
V	63	91
Zn	4420	720
La	13	26
Rb	43	53
Ti	3000	490
Ce	21	47
Cs	1.8	2.1
Sc	8.8	11.5
Hg	2.25	3.25
Pb	36	33

### Estimation of horizontal and vertical mass flux by COMSALT

**Fig. 8:**
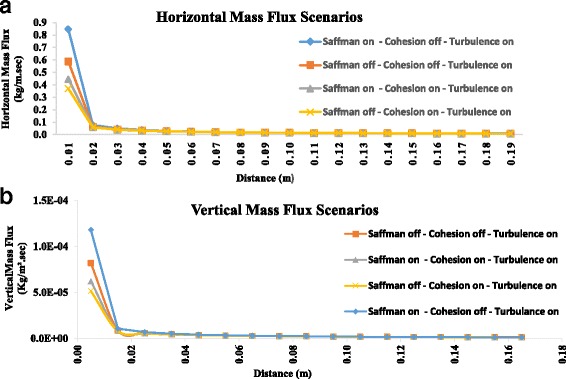
Comparison of calculated **a** Horizontal and **b** Vertical Mass Fluxes using COMSALT model by considering Cohesion, Saffman forces and turbulence

The study area is about 38 Km × 38 Km and is located in the South-West of Iran in Khoozestan province. By considering prevail average wind speed in last 60 years (u = 18.72 Km/h) with dominate percent of 36%, and according to the capabilities of numerical model COMSALT, horizontal and vertical particle mass fluxes are measured for Shalamcheh in various situations. The initial soil and wind parameters are set for a case study according to the physical properties of soil samples and local metrological parameters (Table 1). As it can be seen in Table 6 and Fig. 8, Saffman lift force due to the shearing flow, has an important contribution in formation of horizontal and vertical particle mass fluxes. Interparticle cohesion forces increases the inhibition of saltation, and considering their effect in model led to reduction in particle mass fluxes. The measured shear velocity was below 0.5 m/s; therefore, neglecting the effect of mid-air collision and electrostatic forces will not underestimate the results.

**Table 6:** Estimated vertical mass fluxes by COMSALT for various conditions

Run No.	Saffman Force	Interparticle Cohesion Forces	Turbulence	Vertical Mass Flux (Mt/yr.ha)
1	Not omitted	Omitted	Not omitted	0.905
2	Omitted	Omitted	Not omitted	0.738
3	Not omitted	Not omitted	Not omitted	0.654
4	Omitted	Not omitted	Not omitted	0.599

## Discussion

### Evaluation of metrological parameters

One of the different parameters affecting the occurrence of dust storms is local climatic conditions, such as temperature and rainfall, and wind velocity; exceeding threshold value results in saltation, suspension, or creeping of sand particles. Regarding the size, shape and density of the sand particles and weather condition, they can be transported over long distances [28]. Correlation coefficient calculations between annual and seasonal dust and sand storm occurrence and meteorological parameters indicated a generally negative correlations to precipitation (−0.5) and relative humidity (−0.7). It is positively correlated to wind speed (+0.6) and temperature (+0.6) [29]. The increasing trend of temperature with the last persistent different levels of drought led to disintegration of top soil layer and vegetation cover. Due to the reduction of rainfall, the soil moisture content as the controlling factor of sand and dust storm occurrence can have remarkably reduction [30]. As a result, the dust and sand storm occurrence increased in the studied area and reached 170 days in 2009. Based on Total Zone Mapping Spectrometer (TOMS) data, the recorded sever and extreme dry conditions in the studied area made it vulnerable dust source with persistent average annual rainfall of under 100 mm [10].

The susceptibility of this area to desertification was shown by study done by Cao and co-workers, which show the extended sand and dust storm origin areas in Iraq border (Fig. 7, Region 8 in [31]).

### Relations among particle Size distribution, OM content and soil consistency

The weak structure of soil (granular) as evidenced by the relatively low OM (0.060%) showed the soil volunteer to wind erosion in this area, which was supported by the similar study reporting an increase in erosion capacity with low OM [32, 33]. On the other hand, non-eroded areas can have more clay and are less responsive to wind erosion. Likewise, high OM leads to less susceptible soil to wind erosion because of the binding effect of OM. Also, compared with the similar studies the high plastic index has been seen in this area with an average of 14%, probably because of the sandy nature of the soil (Table 1), which leads to easily erosion [32].

### Mineralogical characteristics of soil and dust samples

The mineralogical composition can indicate regional geology and wind- transported dust, deposited in local soils [34]. According to the Table 4, the soil samples are mostly constituted of SiO_2_. More specifically, mean major elements of soil indicate a predominant SiO_2_ mass component (29.2%) with significant CaO (17.8%) and MgO (6.3%) contributions; a little percent of Al_2_O_3_ (4.9%), Na_2_O (1.82%), K_2_O (1.12%) and total iron as Fe_2_O_3_ (4.19%), as well as the trace amounts (<1%) of V_2_O_5_, TiO_2_, P_2_O_5_, Cr_2_O_3_, NiO,Co_3_O_4_, MnO, SrO, Rb_2_O, CuO, ZrO_2_ and Y_2_O_3_. Compared with various mean shale analyses [33], the Shalamcheh soil is remarkably depleted in Al_2_O_3_, SiO_2_, K_2_O, Na_2_O and Fe_2_O_3_. It is also significantly enriched in MgO and CaO. Emphasizing the dust minerology over Khoramshahr city, can be seen that the airborne dust mainly consists of Quartz, which is dominant component for all dust samples (with average amount of 31.9%). Na_2_O and MgO are the second main mineralogical constituents of dust samples over the studied area with mean mass percentage of 20.9 and 17.7%, while CaO contributes 10.1% and Al_2_O_3_, 6.1%. On the other hand, the soil samples exhibit a lower average percentage for SiO_2_ and higher percentage for Fe_2_O_3_, CaO, TiO_2_ and MnO in comparison with the airborne dust. The distance between the source area and where the dust is deposited can also influence the mineralogical and chemical composition of dust and particle size distribution [2, 34]. The knowledge of the mineralogical composition of wind erodible area is necessary for estimating the composition of emitted dust. It is also necessary for estimating possible impacts on human health, quantitative climate modeling, precipitation, weathering phenomena and ocean biogeochemistry. Chemical composition of dust before its deposition can be altered by atmospheric chemical reactions [2, 35]. Therefore, the mineralogy of the wind eroded soil may be different from obtained results of emitted airborne dust. Considering, much dust storm material is silt-sized quartz, and it, likely causes non- occupational silicosis developing in human lungs if inhaled over a sustained period [2].

### Anthropogenic pollutants of dust and soil samples regarding contamination assessments

In airborne dust samples, Br, Mo, S, Zn and Hg had EF values higher than 10, derived from non- crustal origins. Also, the calculated EFs values for major and trace elements detected in soil samples show Br, Cl, Mo, S, Zn and Hg are of anthropogenic origins in this area (Table 5). One of the important anthropogenic origins of trace elements in this area is Iraq-Iran war remains. High- detected values of Cl and S in the case study can be as the result of using chemical warfare (Mustard gas) in Iraq- Iran war during 1980–1988 [36, 37]. Also, published studies showed the presence of Pb, Cu, Hg, Br, Mo and Zn in contaminated soils because of the intense war activities [38]. The higher value of Hg in the location can be explained by war activities, representing major concern. Also, it can be washed by atmospheric precipitation and make underground water contaminated [37]. The mean measured value of uranium in this area does not differ from background values. Therefore, it can be concluded that there was no unreported use of missiles with depleted uranium. In assessing these data, an additional problem was the fact that no similar papers have been published in this region, and the fact that the data linked to the military industry was difficult to access. Furthermore, any compositional information on wind erodible soils, as reported herein, may help to learn association between observed medical responses and massive dust fall in populated areas and to consider the design of laboratory experiments on health effects of airborne dust [39, 40].

### Horizontal and vertical mass flux estimation by COMSALT

Saltation and sandblasting are the major processes causing particles entrainments and dust producing from soils. To be a major origin of dust, a soil should consist of enough both sand particles to start the saltation mechanism and fine particles such as clays and silts could be transported over long-range distance and local factors such as: the particle size distribution of soils, climate (wind speed and soil moisture content) and surface roughness are controlling the dust emission capacity of soil [41]. The saltating particle motions are calculated mainly by fluid and gravitational forces. In this model the electrostatic forces and also mid-air collisions were omitted affecting particle trajectories mainly for large wind shear velocities. The most important fluid force affecting particle trajectories was the drag force. Also, saltating particles were affected by lift forces including Saffman, due to the shearing flow, and Magnus force through the particle rotation. Horizontal mass flux (mass per unit distance perpendicular to the wind per unit time) mainly consists of saltators with diameters from 20 to 500 μm. Vertical dust flux (mass per unit area per unit time) is comprised of particles with diameters <20 μm that are transported by suspension [24]. Regarding to the particle size distribution of soil samples, around 12% of total particles enters suspension phase and the rest of the particles entering saltation and creeping phase may result in smaller particles because of splashing particles. As it can be seen in Table 6, Saffman forces play an important role in annual vertical mass fluxes in studied area. Also, the lack of interparticle cohesion forces, which can be because of low OM and soil moisture contents, results in considerable annual vertical mass ejection. The researchers showed a direct relation between volumetric soil moisture content and saltation number and dust concentration, indicating a significant reduction in saltation number and dust concentration due to high soil moisture contents [42]. Beside soil moisture content, non-erodible roughness elements such as rocks, pebbles and vegetation can influence the saltation fluid threshold. Roughness elements decrease the wind shear stress on the intervening bare soil and elevate the total threshold wind stress which is needed to start saltation and dust emission [37]. Moreover, observed reduction in horizontal mass fluxes (Fig. 8) are because of particle concentration adjusting [24]. Since the calculated vertical mass fluxes by COMSALT model could be used as emission data in weather forecasting models, producing good information about dispersion, dry and wet deposition of emitted dust could help governments to identify possible affected areas and develop needed policies to reduce possible damages to human health, environment and economy.

## Conclusion

For the first time, the estimated vertical dust fluxes through the COMSALT, soil consistency and variation in regional metrological parameters show the susceptibility of this area (Shalamcheh) to wind erosion. The results indicated the changes in metrological parameters in Shalamcheh located in South –West of Iran. There is an obvious increase in annual mean average Maximum and remarkable reduction in the amount of annual rainfall and the average mean relative humidity content. The strong correlation was seen between changes in metrological parameters and sand and dust storm occurrence in the studied area during the last decades. An extreme dry condition was shown by RDI_st_ estimation as a drought index. The vertical estimated mass fluxes in different conditions, using COMSALT model showed an important role of cohesion forces in inhibition of saltation. The results of models were confirmed by soil consistency tests due to its weak structure, which was expected. The chemistry and mineralogy compositions of the airborne dust samples taken in Khoramshahr city were almost the same and quiet similar to the soil samples collected at several locations downwind. According to the contamination assessments of dust and soil samples, some of these trace elements had EF values higher than 10, suggesting their anthropogenic sources because of the human activities. One of the major non-crustal sources of these trace elements could be the remains of the Iraq-Iran war. Regarding the high vulnerability of this area, wind rose plots and the direction of the dominant blown winds, it is reasonable to conclude that carrying this harmful and contaminated soil by wind can affect the areas, which are far from this region including agriculture lands, would endanger human health. Due to the spread of this crisis to the rest of the world even European countries, it is necessary to identify hotspots, measure their wind erosion capacity and take the effective and functional stabilizing methods of highly erodible regions immediately.

## Abbreviations

AAS: Atomic absorption spectrophotometer
Cd: Contamination degree
Cf: Contamination factor and degree of contamination
COMSALT: Comprehensive model of steady state saltation
EF: Enrichment factor
EPA: Environmental Protection Agency
I-geo: Geo accumulation index
OC: Organic carbon content
OM: Organic matter content
RDI: Reconnaissance Drought Index
RDI_st_: Standardized Reconnaissance Drought Index
USDA: United State Department of Agriculture
XRD: X-ray diffraction
XRF: X-ray fluorescence
